# Shc depletion stimulates brown fat activity *in vivo* and *in vitro*

**DOI:** 10.1111/acel.12267

**Published:** 2014-09-26

**Authors:** Alexey Tomilov, Ahmed Bettaieb, Kyoungmi Kim, Sunil Sahdeo, Natalia Tomilova, Adam Lam, Kevork Hagopian, Michelle Connell, Jennifer Fong, Douglas Rowland, Stephen Griffey, Jon Ramsey, Fawaz Haj, Gino Cortopassi

**Affiliations:** 1 Department of Molecular Biosciences, UC DavisDavis, CA, 95616, USA; 2 Department of Nutrition, UC DavisDavis, CA, 95616, USA; 3 Department of Public Health Sciences MED, UC DavisDavis, CA, 95616, USA; 4 Center for Molecular and Genome Imaging, UC Davis Genome CenterDavis, CA, 95616, USA; 5 Comparative Pathology Laboratory, Department of Pathology, Microbiology and Immunology, VET MED, UC DavisDavis, CA, 95616, USA

**Keywords:** brown adipose, brown adipose tissue, energy expenditure, healthy aging, insulin, Shc

## Abstract

Adipose tissue is an important metabolic organ that integrates a wide array of homeostatic processes and is crucial for whole-body insulin sensitivity and energy metabolism. Brown adipose tissue (BAT) is a key thermogenic tissue with a well-established role in energy expenditure. BAT dissipates energy and protects against both hypothermia and obesity. Thus, BAT stimulation therapy is a rational strategy for the looming pandemic of obesity, whose consequences and comorbidities have a huge impact on the aged. Shc-deficient mice (ShcKO) were previously shown to be lean, insulin sensitive, and resistant to high-fat diet and obesity. We investigated the contribution of BAT to this phenotype. Insulin-dependent BAT glucose uptake was higher in ShcKO mice. Primary ShcKO BAT cells exhibited increased mitochondrial respiration; increased expression of several mitochondrial and lipid-oxidative enzymes was observed in ShcKO BAT. Levels of brown fat-specific markers of differentiation, UCP1, PRDM16, ELOVL3, and Cox8b, were higher in ShcKO BAT. *In vitro*, Shc knockdown in BAT cell line increased insulin sensitivity and metabolic activity. *In vivo*, pharmacological stimulation of ShcKO BAT resulted in higher energy expenditure. Conversely, pharmacological inhibition of BAT abolished the improved metabolic parameters, that is the increased insulin sensitivity and glucose tolerance of ShcKO mice. Similarly, *in vitro* Shc knockdown in BAT cell lines increased their expression of UCP1 and metabolic activity. These data suggest increased BAT activity significantly contributes to the improved metabolic phenotype of ShcKO mice.

## Introduction

Obesity is epidemic in the United States; 34% of adults suffer from obesity. A major consequence is type II diabetes, which is also common in the U.S., and 27% of adults over 65 suffer from this problem. The consequences and comorbidities of obesity and diabetes have a massive negative impact on the aged, including increased risk for cardiovascular disease, cancer, and stroke (Ramirez-Lassepas, 1998; Wilson & Kannel, 2002; Whitmer *et al*., 2008; Yosefy, 2008; Penninx *et al*., 2009; Sakurai *et al*., 2010). Thus, strategies to combat obesity and diabetes are urgently needed and could have a major beneficial impact on the aging population of all developed counties.

Brown fat stimulation as an anti-obesity strategy lagged for several years, because it was thought that brown fat was developmentally eliminated after infancy in humans. More recently, it was shown that adult humans retain differentiated brown adipose tissue depots, (Cypess *et al*., 2009; Virtanen *et al*., 2009) fostering renewed enthusiasm that brown fat activity could be stimulated in adult humans as an anti-obesity therapy.

Brown adipose tissue generates heat via mitochondrial uncoupling (Farmer, 2008) and is recognized for its anti-obesity properties: more active brown fat in humans is associated with good health, whereas less active brown fat is associated with adiposity and insulin resistance (van Marken Lichtenbelt, 2011; Vijgen *et al*., 2011; Cannon & Nedergaard, 2012). Also, rodents with higher amounts of BAT gain less weight and are protected from diabetes (Xue *et al*., 2007), as are rodents that are pharmacologically stimulated for transdifferentiation of WAT depots into brown fat (Ghorbani & Himms-Hagen, 1997).

ShcKO mice were proposed as a model for longevity and healthy aging (Migliaccio *et al*., 1999) over 15 years ago, and they are lean, insulin sensitive, glucose tolerant (Berniakovich *et al*., 2008; Ranieri *et al*., 2010; Tomilov *et al*., 2011), and stress-resistant (Napoli *et al*., 2003; Camici *et al*., 2007; Menini *et al*., 2007; Martin-Padura *et al*., 2008). While ShcKO mice have deletion of p66Shc, they also exhibit a significant reduction of p52/46Shc isoforms in insulin-responsive tissues (Tomilov *et al*., 2011). As Shc has been implicated in adipocyte differentiation (Laurino & Cordera, 1998; Boney *et al*., 2001; Woldt *et al*., 2011), we investigated effects of Shc reduction on brown adipose properties in ShcKO mice.

## Results

### BAT is more active in ShcKO than in control mice

Shc functions *in vivo* and *in vitro* as inhibitor of metabolic insulin signaling, and thus, ShcKO mice exhibit improved insulin sensitivity in peripheral tissues and improved systemic glucose tolerance (Tomilov *et al*., 2011). To determine which tissues contribute to metabolic phenotype of ShcKO mice, we performed a kinetic measurement of glucose uptake *in vivo* using positron emission/computer tomography (PET/CT). Mice were challenged with insulin in the pseudofed (i.e. pre-injected with glucose) (Fig.1a–f) state. In the pseudofed state, radiotracer uptake by interscapular BAT and gastrocnemius muscle was obviously and significantly higher in ShcKO mice compared with controls (Fig.1a,b). PET intensities from liver, muscles (gastrocnemius and quadriceps femoris group of muscles), epididymal fat, kidneys, heart and BAT tissue were plotted as a function of time and analyzed. Uptake of FDG was strongly insulin-dependent; tissues with the largest insulin response were BAT, liver, muscle, and heart (Figs1c,d and S1), which had faster radiotracer uptake kinetics in ShcKO than controls. Much lower FDG accumulation was observed in WAT before or after insulin stimulation (data not shown). Difference between ShcKO and control mice was significant for only BAT (Fig.1e), muscles (Fig.1f), heart, and liver (Fig. S1).

**Figure 1:**
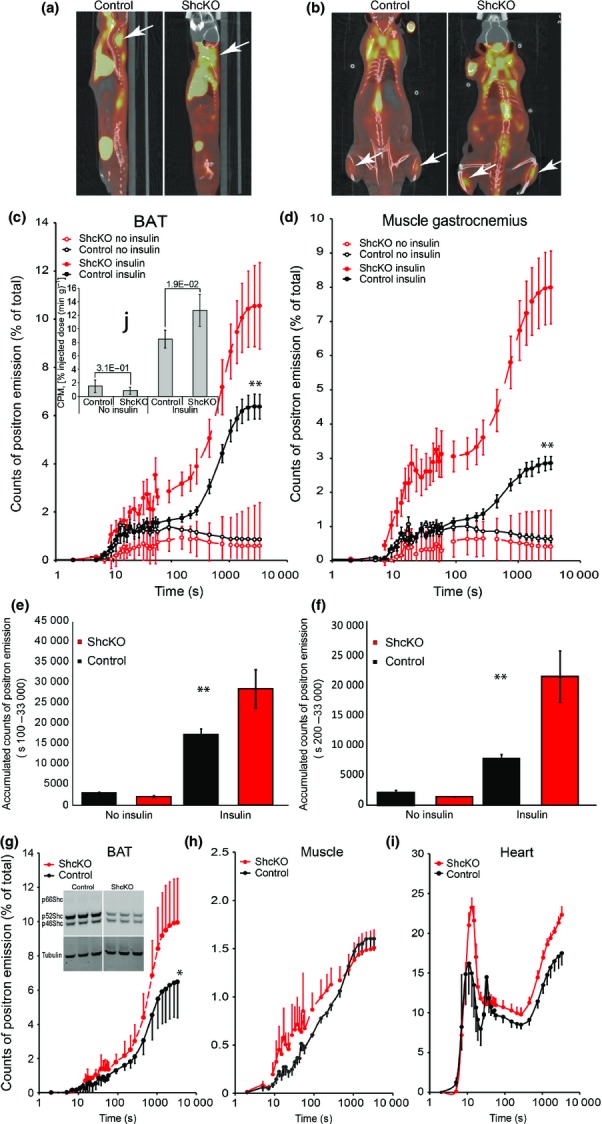
(a, b) Sagittal and axial PET/CT images of pseudofed mice through brown adipose tissue (BAT) and gastrocnemius muscle, shown by arrows. (c, d) PET intensities were normalized to total positron emissions for each mouse and plotted against time, traces for BAT and muscle are presented. (e, f) bars are means and SE of area under the curve (AUC) for BAT and muscle, *P*-values were generated with ANOVA, n = 4. (g, h, i) Fasted mice were stimulated with insulin. Traces of the [^18^F]FDG uptake by BAT, muscles and heart are shown. Reduction of Shc expression in ShcKO BAT is shown on Western blot, *P < 0.1, **P < 0.05.

Endpoint bio-distribution (EPBD) measurements also confirmed an increased FDG uptake only in interscapular BAT. In EPDB, mice were analyzed for radiotracer accumulation in 15 dissected tissues: muscles (three different groups), liver, kidneys, interscapular BAT, WAT (epididymal, visceral, perirenal, and subcutaneous) skin, bone marrow, urine, small intestine, and brain by scintillation counting, revealing similar trends as in the PET/CT study; however, only ShcKO BAT showed a significant increase (*P* = 1.9E-02) in insulin-dependent FDG uptake compared with controls (Fig.1j). Thus, by PET-CT pseudofed analysis and also EPBD analysis, there is a strong and significant increase in BAT metabolic activity.

In the fasted state, there was no difference in insulin-dependent FDG uptake by any tissues (Fig.1g–i). However, ShcKO BAT accumulated 9.88% ± 1.3 of total FDG, whereas control BAT accumulated only 6.39% ± 1.2. Thus, ShcKO BAT was accumulating about 1.5-fold more radiotracer than controls although not statistically significant (*P* = 9.03E-2) with four animals per group and under alpha 5%.

### ShcKO BAT is more metabolically active *ex vivo* than controls

We excised interscapular BAT, dissociated the cells, and measured their mitochondrial physiology on the Seahorse. Primary ShcKO BAT cells had three times higher basal mitochondrial O_2_ consumption rate (OCR) than controls, suggesting an increased flux through glycolysis and the TCA cycle in ShcKO BAT. Oligomycin-inhibited OCR was 3.9 ± 2.67 times higher than controls, attributable to increased uncoupling (Nicholls *et al*., 2010). FCCP-stimulated respiration, which reveals the maximal respiratory capacity, of ShcKO BAT was also higher than controls (Fig.2a,b).

**Figure 2:**
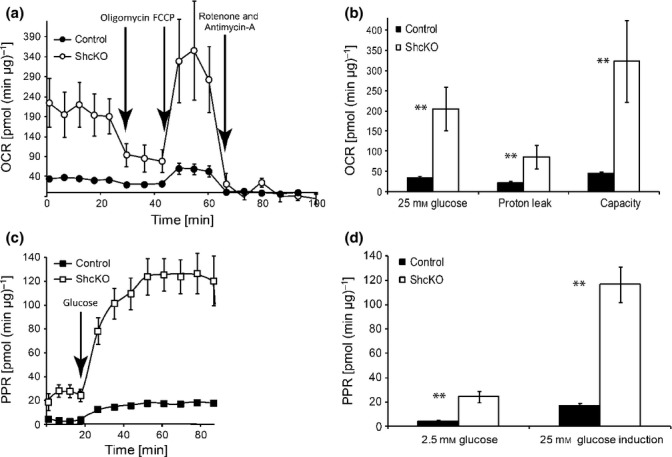
(a, b) Standard bioenergetic profiling traces and bars of O_2_ consumption rate (OCR) of ShcKO and control mice brown adipose tissue (BAT) cells cultivated in 25 mm glucose and 20% FBS, regiments are indicated, *P*-values were generated by AUC ANOVA n = 8. (c) traces of GLUCOX assay, BAT cells were ‘fasted’ in 2.5 mm glucose for 16 h. Rates 1–4 are basal proton production rate (PPR). Media was supplemented with 25 mm glucose as indicated, and PPR was recorded for additional 8 cycles. (d) Bars are means and SD of PPR, n = 8, and *P*-values were generated by AUC ANOVA. Two–three mice were used per day in three independent experiments, results were grouped by genotype; technical replication was 5, *P < 0.1, **P < 0.05.

The proton production rate (PPR) is a measure of glycolytic activity (Nicholls *et al*., 2010). Primary BAT cells were ‘fasted’ and then given glucose, to mimic the fasted-to-fed transition. At exposure to glucose, PPR was gradually and significantly increased in both ShcKO and control brown adipocytes; however, the rate was much faster in ShcKO brown adipocytes (Fig.2c,d).

### ShcKO mice have increased expression of major metabolic enzymes in BAT

We measured the expression of several important metabolic enzymes in BAT, liver and muscles of ShcKO and controls, and in shRNA, Shc knockdown differentiated mouse brown fat cell line (BATCL). Results are presented in Table S1. The most relevant blots are presented in Figs3a (BAT) and S2a (muscles) along with densitometry and statistical analysis, in Figs3b–e*,* and S2b. Full gel images for ShcKO and control BAT Western blots are on Fig. S3. Only ShcKO BAT exhibited an increase in UCP1 and UCP3 levels. Moreover, fifteen metabolic enzymes were significantly upregulated only in BAT of ShcKO, among them such important enzymes governing entrance to glycolysis and flux to mitochondria: HK, GCK, and PDH. Phosphorylation status of FoxO3a was increased (Table S1), and expression of PDK4 (a repressor of mitochondrial PDH complex) was decreased. Expression of several enzymes of the beta-oxidation cascade were increased in ShcKO BAT, including CPT2, ACADVL, MCAD, ACAA2, and ETFA (Fig.3a,c*,* Table S1). These results indicate that glycolysis, and mitochondrial pyruvate, and fat oxidation are increased in ShcKO BAT. By contrast, there was no consistent upregulation of metabolic enzymes in muscle or liver of ShcKO mice. There was no significant change in VDAC (Figs3 and S2, Table S1) or mtDNA/nuclear DNA ratio (data not shown) in any tissue.

**Figure 3:**
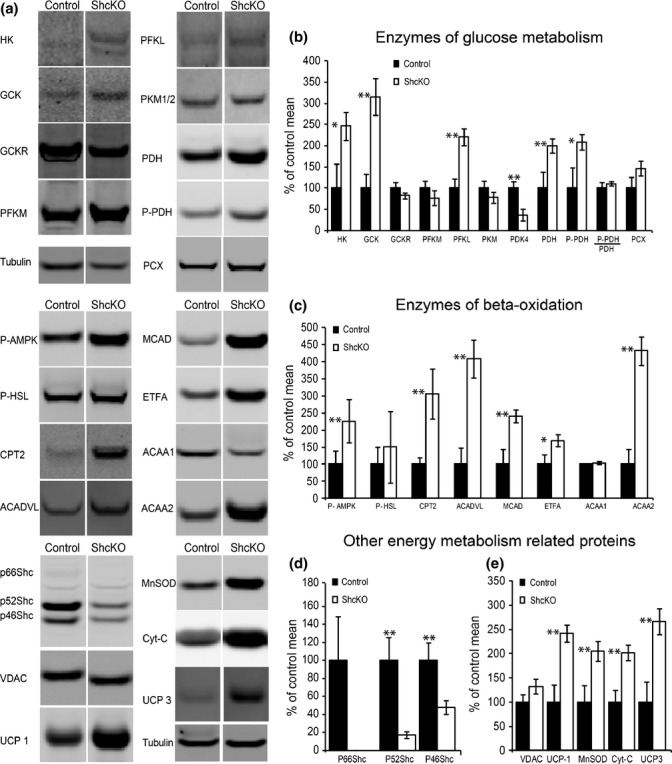
(a) Representative Western blots are presented. Mice were kept in a conventional animal facility, fasted for 6 h and anesthetized with Nembutal 100 mg kg^−1^, the brown adipose tissue (BAT) tissue was extracted and Western-analyzed with indicated antibody. Signals were corrected by tubulin and presented as % of control, bars are means and SD. (b), expression of enzymes related to glycolysis, n = 5; (c) beta-oxidation, n = 5, (d) Shc isoforms, n = 20; (e) other energy metabolism related; *P*-values of ShcKO versus control were determined by two-tailed *t*-test, *P < 0.1, **P < 0.05.

We tested whether white–brown transdifferentiation ‘browning’ of other white fat depots occurs in ShcKO mice by quantifying BAT-differentiation markers and UCP1 expression in WAT, liver, and muscles, but did not find any sign of increased BAT-like differentiation in ‘non-BAT’ tissues. By contrast, expression of BAT-differentiation-specific markers was higher in ShcKO BAT than CTL BAT (see 2 paragraphs below).

### ShcKO BAT consists of smaller cells

At the level of cellular morphology, there was a significant increase in number of smaller brown adipocytes (*P* < 0.05), and a significant decrease in the number of larger brown adipocytes (*P* < 0.05) in ShcKO BAT than controls (Fig.4a); statistics used are described in figure legend and Methods. Additionally, lipid droplets were smaller in ShcKO BAT (Fig.4b), and this is consistent with Oil Red O staining shown on Fig. S4, in which, Shc was silenced in mouse brown adipocyte cell line BATCL, and cells were differentiated into a mature adipocytes. Though there was a reduction in cell size of ShcKO BAT, there was no reduction in total BAT mass (Fig.4c). At 18 months of age, all tissues were analyzed for cellular morphology by a very experienced laboratory animal pathologist. The only tissue with altered cell morphology identified by the pathologist was brown fat.

**Figure 4:**
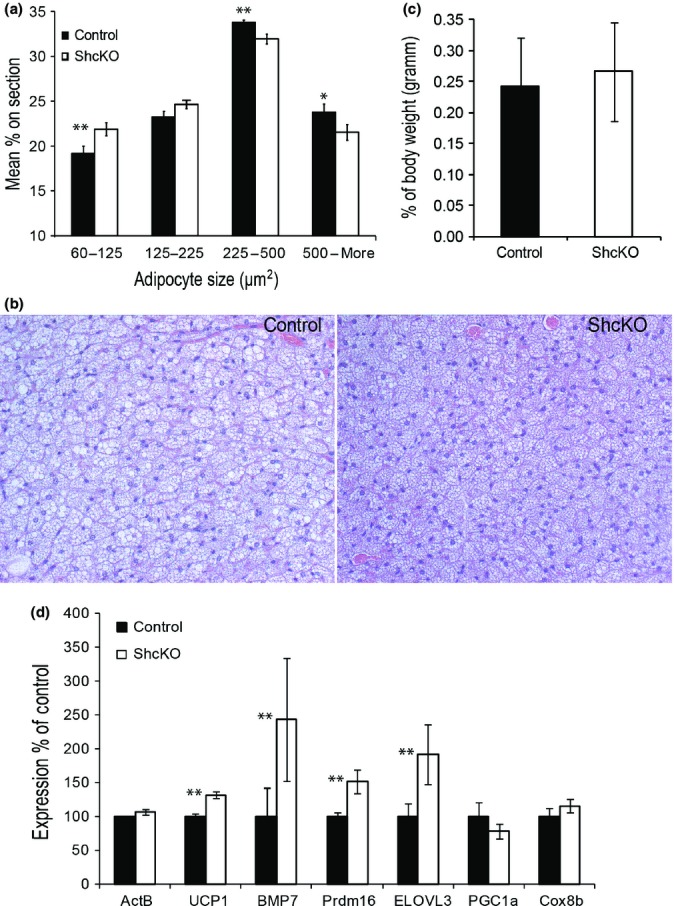
(a) Bars are % of adipocytes falling in indicated size distribution, *P*-values were calculated by nested random effect model, n = 5, technical replication also 5. (b) Representative image of control and ShcKO brown adipose tissue (BAT) sections at 250×. (c) Bars are means and SD of ShcKO and control interscapular BAT weights expressed as % of body weight, n = 6. (d) Mice were housed at 21 °C, interscapular BAT was extracted and expression of differentiated BAT markers was measured with Q-RT–PCR. Bars are means and SD, values are expressed as % of control, n = 4, technical replication 3, and *P*-values generated by One Way ANOVA followed by Scheffe's post hoc analysis, *P < 0.1, **P < 0.05.

The BAT-specific markers UCP1, BMP7, Prdm16, PGC1a, ELOVL3, and Cox8b are diagnostic for differentiated BAT tissue (Ahfeldt *et al*., 2012). There was no increase of BAT markers expression in any depot of ShcKO WAT; that is, no increased transdifferentiation of white adipocytes into brown was observed (data not shown). However, in ShcKO BAT, expression of four out of six markers of differentiated brown adipocytes was increased: UCP1, Prdm16, BMP7, and ELOVL3, *P* = 1.8E-02, 1.1E-03, 3.0E-02, and 2.2E-02, respectively (Fig.4d).

### Inhibition of BAT abolishes the improved ITT/GTT of ShcKO mice

ShcKO mice have been shown to be more insulin sensitive and glucose tolerant than controls under standard conditions by three groups (Berniakovich *et al*., 2008; Ranieri *et al*., 2010; Tomilov *et al*., 2011) Fig.5a,b. However, when the mice were pre-injected with an alpha-adrenergic BAT-specific inhibitor SR5930A (Ootsuka *et al*., 2011), the difference between the mutant and control mice was abolished (Fig.5c,d). Similar results were observed with Nembutal, which is less specific for inhibition of brown fat, but is known to suppress brown fat sympathetic innervation (Shimokawa *et al*., 1998).

**Figure 5:**
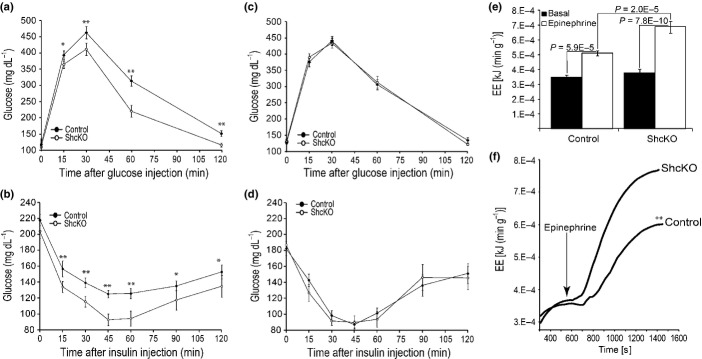
(a) GTT, (b) ITT of ShcKO and controls; (c) GTT, (d) ITT, mice were pre-injected with SR5930, n = 15. (e) Bars are means and SE of basal and epinephrine-induced energy expenditure (EE) of ShcKO and control mice housed at 21 °C, n = 9. (f) Traces are representative epinephrine-induced EE curves of control and ShcKO mice as indicated. *P*-values were generated by one way ANOVA followed by Scheffe's post hoc planned comparison, *P < 0.1, **P < 0.05 for ShcKO versus control or indicated as face values.

### ShcKO mice have increased epinephrine-induced energy expenditure that is abolished by increased housing temperature

Control and ShcKO mice were acclimated to either 21 °C or 28 °C (thermoneutrality) for 1 week and stimulated with epinephrine during indirect calorimetry measurement. Shortly after the BAT stimulation with epinephrine, the energy expenditure (EE) increased and stabilized at a new higher level. When mice were acclimated to 21 °C (Fig.5e,f), the basal EE was similar (*P* = 2.2E-01) between ShcKO 3.8E-4 ± 1.7E-5 kJ per (min*g) and control 3.5E-4 ± 1.3E-5 kJ per (min*g); whereas epinephrine-induced EE was 5.1E-4 ± 2.5E-5 kJ per (min*g) for control and 6.9E-4 ± 4.1E-5 kJ per (min*g) for ShcKO (*P* = 2.0E-05). At 28 °C, there was no difference between genotypes (data not shown).

IL-6 is involved in BAT-mediated whole-body energy metabolism and improvement of ITT/GTT after BAT transplantation (Stanford *et al*., 2013). We measured but did not find a significant difference between ShcKO and control mice IL-6 levels, Fig. S5.

### Shc effects on BAT are cell autonomous

To test whether Shc effects are a cell autonomous, we generated brown fat pre-adipocytes with a stable knockdown of Shc and differentiated them into mature brown adipocytes. shShc knockdowns had decreased Shc expression (Fig.6a), and insulin-mediated inhibitory phosphorylation of FoxO3a was increased. We analyzed the expression of 40 metabolic enzymes (Table S1). The expression and activity status of PDH (Fig.6b–d) was increased. There was a marked increase of UCP1 expression, (UCP1 is the main mitochondrial uncoupling protein) and increased ETFA expression (beta-oxidation enzyme) was observed. Just as in ShcKO BAT tissue, shShc BATCL showed threefold increased respiration on 25 mm glucose, and an increased respiratory capacity compared with shControl (Fig.6e). ‘Fat-fueled’ (palmitate/BSA) shShc BATCL also consumed more oxygen than shControls (Fig.6f). Thus, inhibition of Shc increased expression of metabolic enzymes in the mouse brown fat cell line BATCL *in vitro*. Once differentiated, the shShc BATCL showed increased expression of UCP1, metabolic enzymes, and increased metabolic activity on both glucose and lipid substrates. Similarly, increased oxygen consumption after p52/46Shc knockdown was observed in mouse liver cells FL83B, mouse muscle cells C2C12, and HeLa cells by two different methods: BD Biosensor Oxygen Detector plates and Seahorse XF-24 extracellular flux analyzer. By contrast, Shc overexpression suppressed metabolic activity of the cell lines (data not shown). No increase in metabolic activity of cells was found, when only p66Shc was knocked down. Thus, Shc effects on BAT metabolism are consistent with cell-autonomous idea; however, it appears that p52/46 Shc reduction is the major contributor to the phenotype.

**Figure 6:**
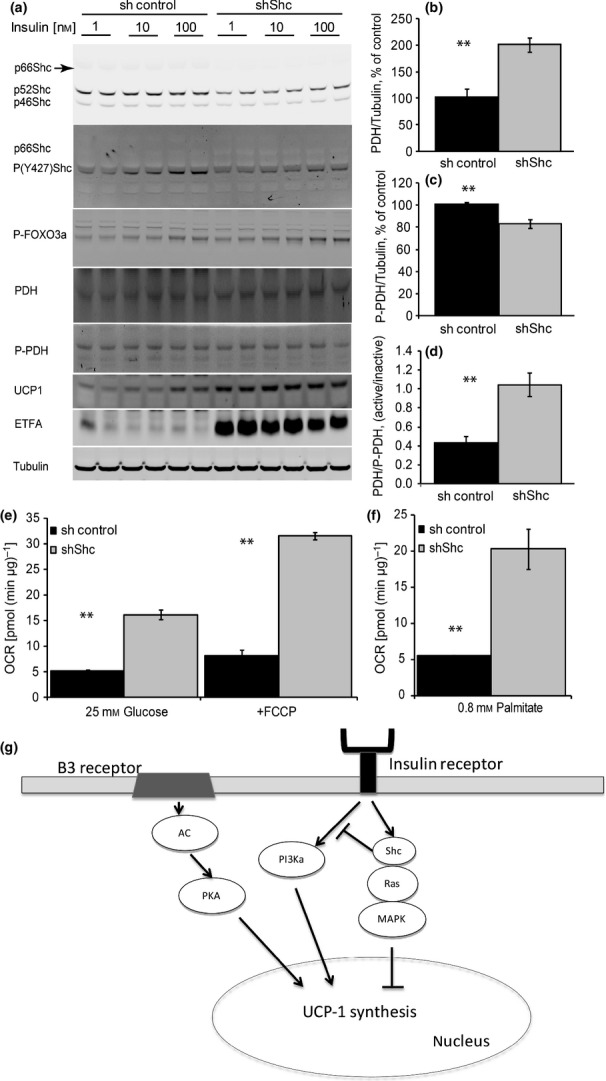
(a) differentiated shShc and shControl brown fat cell line (BATCL) stimulated with insulin and Westerns analyzed with indicated antibody. (b, c, d) bars are densitometry means and standard deviations of PDH expression normalized to tubulin, total PDH, phospho PDH (P-PDH), and ratio PDH/P-PDH, respectively, *P*-values were from two-tailed *t*-test, n = 6. (e, f) OCRs of shControl and shShc BATCL utilizing 25 mm glucose or 0.8 mm palmitate, as indicated, and *P*-values are determined with AUC ANOVA, n = 10. (g) Model of regulatory elements for UCP1 expression adopted and modified from (Klein *et al*., 2000). UCP1 expression is regulated both by G protein-coupled β3-adrenergic receptors and signals from the insulin receptor. Shc transfers insulin signal to ERK-the inhibitory arm of UCP1 expression. Shc opposes Pi3Kα arm of UCP1 regulation the stimulatory arm. Thus, Shc reduction makes the inhibitory arm weaker and stimulatory arm stronger, and promotes UCP1 expression*P < 0.1, **P < 0.05.

## Discussion

### ShcKO mice as a model of healthy aging

The p66ShcKO mouse has been used since 1999 as a model of healthy aging and longevity; originally its beneficial traits were attributed to redox properties of p66Shc (Migliaccio *et al*., 1999). Shc-depleted (ShcKO) mice resist obesity on chow diets and on high-fat diets and are more insulin sensitive and glucose tolerant at young and old age (Berniakovich *et al*., 2008; Ranieri *et al*., 2010; Tomilov *et al*., 2011). Thus, Shc mice are a model for anti-obesity and antidiabetes mechanisms and to the extent that these decline with age, ‘healthy aging’. ShcKO mice also resist multiple stresses and injury, including ischemia reperfusion injury.

Recently, we showed that resistance to high-fat diet, increased insulin sensitivity and glucose tolerance of these mice are results of changes in expression of major Shc isoforms: p52 and p46Shc rather than p66Shc deletion. We compared two different p66Shc knockouts made in slightly different ways by the T. Prolla group and the PG Pelicci group. The p66Shc mouse made by the Prolla group has precise deletion of p66Shc only, this mouse has no health benefits, no increased insulin sensitivity and glucose control, and no obesity resistance (Tomilov *et al*., 2011). By contrast, in the mouse made by the Pelicci group, perhaps as a result of the residual unexcised neomycin cassette in addition to the p66Shc deletion, there is decreased expression of p52/p46 in insulin-responsive tissues and increased expression of p46 in WAT (Fig. S6). Comparison of Pellici ShcKO to T. Prolla generated p66ShcKO rules out the p66Shc isoform, and thus, the beneficial health effects of Shc depletion are the result of p52/46 depletion in ShcKO mice rather than p66Shc deletion. We demonstrated earlier, that p52/p46 Shc functions as a repressor of metabolic insulin signaling and that in animals, tissues, and cells p52/p46 Shc depletion stimulates insulin signaling (Tomilov *et al*., 2011). Furthermore, p52:p46:p66 ratios are 100:50:1 in tissues. Also, depletion of p66Shc in cells has no metabolic consequences, whereas p52/46 depletion in cells causes increased insulin sensitivity and mitochondrial activation. Thus, it is the decrease in the major Shc isoforms p52/p46, rather than deletion of the minor Shc isoform, which drives both animal adiposity and insulin sensitivity phenotypes, and cellular mitochondrial and insulin sensitivity phenotypes.

To further specify the mechanism of reduced adiposity of ShcKO mice, we tested the insulin sensitivity of ShcKO WAT and found it to be less sensitive, providing one possible mechanism for reduced adiposity (Tomilov *et al*., 2011). However, ShcKO mice are overall more insulin sensitive at the whole-body level, so we carried out PET/CT experiments to determine which tissues were specifically contributing to the insulin sensitivity and thus identified the BAT as a major contributor.

### Brown fat is an important force for reduced adiposity in ShcKO mice

As we imaged the consequences of insulin administration in ShcKO mice, we noticed an especially strong glucose uptake in brown fat (Fig.1), the only significant difference among all tissues surveyed in both fed and fasted state. Thus, BAT appears to be a sink for nutrient excess. Under pseudofed conditions to mimic fasted/fed transition, muscles were also more metabolically active and may partially contribute to the ShcKO. However, there are multiple reasons that muscle is not the major contributor to the increased insulin sensitivity of ShcKO mice. First, pharmacological BAT inhibition with two different drugs abrogated the ShcKO metabolic benefit. Second, thermoneutrality (Fig.6g), which decreases the BAT-specific UCP1 expression and BAT activity also abrogated the ShcKO metabolic benefit. Third, by EPBD of radioactive glucose uptake, *only* ShcKO BAT is significantly more active; no other tissues have significant changes, and under fasted conditions only BAT showed a difference. Fourth, muscle and liver glycolytic enzyme expression and activity were not increased, and in fact, some were decreased under fasting conditions, suggesting that muscle and liver are not important sinks of glucose in ShcKO mice (Fig. S2, Table S1, K. Hagopian, A.A. Tomilov, K. Kim, G.A. Cortopassi and J.J. Ramsey submitted). Fifth, we have carried out extensive pathological analysis of 100 s of ShcKO mice by laboratory animal pathologists at several age points, and the only recognizable cellular morphological differences were in BAT.

### How does BAT contribute to the metabolic phenotype of ShcKO mice

Once focused on BAT, we find several mechanisms operate to activate them in ShcKO mice. First, BAT is more ‘brown’, that is more differentiated, with higher expression of UCP1 that uncouples mitochondria, making them more ‘leaky’, expending more energy (Berniakovich *et al*., 2008), and multiple differentiation markers are elevated in ShcKO BAT. Second, as a consequence of this gene expression and mitochondrial uncoupling, there is increased mitochondrial physiological oxidative capacity in ShcKO brown adipose cells *ex vivo* (Fig.2). Third, this increased activity, demonstrated physiologically, is supported by increased expression of lypolytic and mitochondrial-limiting enzymes (PDH, ETFA, and ACAA2) (Fig.3). Fourth, Shc knockdown in BAT cell lines (Fig.6) increases PDH, ETFA, ACAA2, and UCP1, showing a Shc-specific effect on mitochondrial activation in this cell type.

### Relevance and relationship of mouse BAT to the Human perspective

Although for many years, BAT was not thought to exist in adults at all, recently, multiple brown fat depots have been identified (cervical, clavicular, paravertebral, mediastinal, para-aortic, and suprarenal), and demonstrated as active ‘sinks’ for glucose *in vivo* and to be important for obesity resistance and insulin sensitivity (Cypess *et al*., 2009; Virtanen *et al*., 2009; Farmer, 2008; van Marken Lichtenbelt, 2011; Muralidhara & Muralidhara, 2011; Virtanen & Nuutila, 2011; Cannon & Nedergaard, 2012). The data presented here suggest that depletion of p46/p52 Shc increases differentiation, enzyme activity, and mitochondrial activity of BAT, which increases insulin sensitivity and glucose tolerance in mice. Thus, genetic or pharmacological Shc inhibition may be a viable strategy to increase brown fat activity in pursuit of anti-obesity or antidiabetic goals. This strategy could have impact in the U.S., and other developed countries in which obesity and diabetes and their comorbid consequences are major heath issues for the aged.

### Conclusions

1. In ShcKO mice brown fat is hyperactivated, contributes to glucose disposal and likely contributes to the healthy phenotype
2. These effects may be cell autonomous and involve signaling roles of major Shc proteins in insulin signaling
3. This work supports the notion that targeting brown fat by Shc inhibition may represent a strategy to prevent human obesity and its detrimental consequences on age-related disease.

## Experimental procedures

### Animals

ShcKO are originally described in (Migliaccio *et al*., 1999). Mice were kept pathogen-free throughout the study. All experimental procedures were approved by the Institutional Animal Care and Use Committee (IACUC) and were performed in compliance with local, state, and federal regulations. Diet was LM-485 (Teklad 7012) with nutrient composition: protein = 19.9%, carbohydrates = 53.7%, fat = 5.7%. The source of lipid was soybean oil, protein – soybean meal, energy source – ground oats.

### Materials and antibodies

Media DMEM and F12-B, minimal essential medium amino acids, penicillin/streptomycin, fetal bovine serum were purchased from GIBCO (Grand Island, NY, USA). Insulin and specific brown fat inhibitor SR59230A were from Sigma (St. Louis, MO, USA). Antibodies were from Cell Signaling Inc. (Danvers, MA, USA) except: rabbit anti-Shc and anti-Cyt-C antibody were from BD Biosciences (San Diego, CA, USA); rabbit antiphospho-PDH, PCX, UCP3, goat anti-GCKR, mouse anti-ACADVL were from Novus Biologicals (Littletown, CO, USA); anti-Tubulin – Sigma; rabbit anti-UCP1 and CPS1, mouse anti-PDH, MCAD, and ACAA1 were from Abcam Inc. (Cambridge, MA, USA); rabbit anti CPT2, PFKL, HK were from Epitomics Inc. (Burlingame, CA, USA); rabbit anti-GCK – from LifeSpan BioScience Inc. (Seattle, WA, USA); goat anti-PFKM were from GenWay Biotech Inc. (San Diego, CA, USA); goat anti-UCP2 were from Santa Cruz Biotechnology Inc. (Santa Cruz, CA, USA); the secondary infrared dye 700 and 800 labeled antibody were from Li-Cor Biosciences (Lincoln, NE). Trans-Lenti shRNA packaging plasmids, shShc-GIPZ lentiviral shRNA and Nonsilencing-GIPZ lentiviral shRNAmir control plasmids were from Thermo Scientific Inc. (Pittsburgh, PA, USA).

### PET/CT measurement of glucose uptake *in vivo*

In pseudofed experiments, mice were injected with 2 mg kg^−1^ of glucose intraperitoneally 15 min prior to the injection of ^18^F-FDG. The mice were anesthetized with a mixture of isoflurane/oxygen and imaged dynamically using a Focus 120 small animal PET scanner (Siemens Preclinical Solutions, Knoxville, TN, USA) with a resolution of 1.3 mm. Five seconds after the start of the acquisition, the animals were administered ∼200 uCi of ^18^F-FDG (PETNET Pharmaceuticals- Sacramento, CA) in addition to 3 mU g^−1^ of body weight of insulin (when insulin was used) via tail vein. Anatomical images were taken with a small animal InveonMM microcomputed tomography (CT) scanner (Siemens Preclinical Solutions) for coregistration with the PET images with corresponding voxel size of 0.097 mm × 0.097 mm × 0.097 mm. PET intensities were exported by Inveon Research Workplace software (Siemens, Marburg, Germany).

### Western blotting

Proteins were isolated with Cell Lysis Buffer (Cell Signaling Technologies), resolved by SDS-PAGE, transferred, blocked with Blocking Buffer (Li-Cor Biosciences), and hybridized with indicated primary and IR-dye 700CW, 800CW secondary antibody.

### Insulin and glucose tolerance tests

Mice were fasted for 6 h and given intraperitoneal injection of insulin (0.2 U kg^−1^). In the glucose tolerance test after 16 h fasting, mice were given injection of glucose (2 mg kg^−1^); the blood glucose was measured. Same groups of mice were used in ITT/GTT, but mice were pre-injected with a specific brown fat inhibitor SR59230A (5 mg kg^−1^) n = 15.

### Bioenergetic profiling

Brown adipocytes were isolated as described (Fasshauer *et al*., 2001), 200 μL per well of cell suspension was plated on 24 well plates (Seahorse Biosciences, Billerica, MA, USA) and incubated overnight. Media was changed to unbuffered DMEM, 20% FBS, 200 mm glutamax, 100 mm sodium pyruvate, 25 mm glucose, pH 7.4. Cells were pre-equilibrated for 1 h; OCR and PPR were recorded with Seahorse XF-24. Regimens: Oligomycin – 1 μm, FCCP – 12.5 μm, Rotenone – 0.1 μm, Antimycin A – 1 μm. Total protein in each well was measured. Values were expressed as pmoles per [min*μg (protein)].

Brown fat cell line (Fasshauer *et al*., 2001) was stably transfected with lentiviral shShc and shNon-silencing-GIPZ shRNA. Stable transfected cells were selected with FACS for GFP. Fifty thousand cells per well were seeded on Seahorse plate and differentiated in differentiation media containing 20% FBS, 20 nm insulin and 1 nm triiodothyronine for 48 h, than were supplemented with 0.5 mm dexamethasone, 0.5 mm isobutylmethylxanthine, and 0.125 mm indomethacin for 48 h, and returned to differentiation media. At day 8 cells were differentiated. Unbuffered DMEM containing 10% FBS was used for glucose driven OCR measurements; KHB-for palmitate driven OCR. KHB: 111 mm NaCl, 4.7 mm KCl, 2 mm MgSO_4_, 1.2 mm Na_2_HPO_4_, 0.5 mm carnitine, 1% FBS, pH 7.4.

### Indirect respiration calorimetry

Energy expenditure was measured in 0.8 L chambers with catheter for subcutaneous injections. Animals were acclimated to 21 °C or 28 °C for 1 week, anesthetized with 100 mg kg^−1^ of Nembutal, catheterized and adapted to the chambers for 20 min, epinephrine was 1 mg kg^−1^. Air was drawn at 250 mL min^−1^. Oxygen content was measured by O_2_ analyzer (FC-10, Sable Systems), and CO_2_ – by CO_2_ analyzer (CA-10, Sable Systems). EE was calculated with Weir equation (Cunningham, 1990):
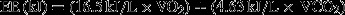


Brown adipose tissue adipocytes size distribution was measured using ImageJ software (NIH, Bethesda, MD, USA) on H&E stained five random 100 μm sick sections of BAT from each of five animals of each genotype in double blinded experiment. Micrographs resolution was 2.826 pixels μm^−1^, 250×.

Primers for Q*-RT–PCR* were as follows: Cox8b CCCAGCTGGCTACCTAAGTG, GAACCATGAAGCCAACGACT, ELOVL3 ATGAACTTTGGCGTCCATTC, CTTTCTCCTGCCTCCAGATG, PGC1a ATGTGTCGCCTTCTTGCTCT, CACGACCTGTGTCGAGAAAA, Prdm16 CAGCACGGTGAAGCCATTC, GCGTGCATCCGCTTGTG, BMP7 CCTGTCCATCTTAGGGTTGC, GCCTTGTAGGGGTAGGAGAAG, UCP1 GATGGTGAACCCGACAACTT, and CTGAAACTCCGGCTGAGAAG.

### Statistical analysis

with sas 9.4 (SAS Institute, Cary, NC, USA) and Excel Statistical Data Analysis Tool package 2007, unless indicated otherwise, was performed with One Way ANOVA followed by Scheffe's post hoc planned comparison test, (*)-*P* < 0.1, (**)-*P* < 0.05. For the ShcKO and controls BAT cell size distribution, we performed nested random-effects analysis of variance for the data with a nested structure, where five animals were randomly chosen in each genotype, and then five slides for each animal were quantified. For uptake of FDG measured at serial time points postinsulin injection, we utilized the area under the PET counts time curve as a measure for the aggregate effect over a period of time. AUC values were calculated using the trapezoidal rule. Then the total AUC values were compared between groups by anova to test the overall effects of the Shc deletion on insulin-dependent FDG uptake.

## Supporting Information

Figure S1 (a, c) PET/CT was performed as described. PET intensities were normalized to total positron emissions for each mouse and plotted against time; traces for heart and liver are presented. (b, d) bars are means and SE of AUC, *P* values were generated with ANOVA, n = 4.

Figure S2 (a) Representative Western Blots are presented. Mice were kept in a conventional animal facility, fasted for 6 h and anesthetized with Nembutal 100 mg kg^−1^, the muscle tissue was extracted and Western-analyzed with indicated antibody. (b) Signals were corrected by tubulin and presented as % of control, bars are means and SD, n = 5; *P*-values of ShcKO vs. control were determined by two tailed *t*-test, (*)-*P* < 0.1, (**)-*P* < 0.05.

Figure S3 Mice were kept in a conventional animal facility, fasted for 6 h and anesthetized with Nembutal 100 mg kg^−1^, the BAT tissue was extracted and Western-analyzed with indicated antibody. C = control, S = ShcKO.

Figure S4 Fully differentiated shShc and shControl BATCL, as indicated, stained with Oil Red-O.

Figure S5 Blood was collected from tail of ShcKO and control mice, 5 month old. IL6 levels were measured with Mouse IL-6 ELISA Kit (Piercenet, Thermo Scientific) Cat#: EM2IL6. Bars are mean and SD, n = 3.

Figure S6 (a) Liver, muscles, pancreatic islands, interscapulat BAT, heart, epididymal WAT, visceral WAT, perirenal WAT, spleen, mouse embryonic fibroblasts (MEFs), macrophages, brains, lungs from ShcKO and control mice were Western analyzed with anti-Shc antibody. Table shows relative expression of Shc isoforms in ShcKO mice to control. n > 5, *P* < 0.05, fold change (FC)>2.0. (b) Representative Western Blots of Shc isoforms expression in ShcKO and control mice tissues, as indicated.

Table S1 BAT, muscle, liver from ShcKO and control mice, and shShcBATCL, and shControlBATCL were western analyzed with indicated antibody. Direction of ShcKO/control fold change is indicated in the table. *P* < 0.05, n = 5. For BATCL n = 6. *P*-values were from two tailed *t*-test.
