# Supplementary material for: Shc depletion stimulates brown fat activity in vivo and in vitro
Source: Aging Cell. 2014 Sep 26;13(6):1049–58. doi: 10.1111/acel.12267 (PMC4244234; doi:10.1111/acel.12267)
Supplement: Supplementary file 1 [file acel0013-1049-sd7.doc]

|  |  |  | **ShcKO/Control** | **shShc/shControl** | **ShcKO/Control** | **ShcKO/Control** |
| --- | --- | --- | --- | --- | --- | --- |
|  | **Gen Symbol** | **Name** | **BAT** | **BATCL** | **Muscle** | **Liver** |
|  | **Glucose oxidation** | |  |  |  |  |
| 1 | GLUT4 | solute carrier family 2 (facilitated glucose transporter), member 4 |  |  | Upregulated |  |
| 2 | GCK | glucokinase | Upregulated | Upregulated | Same | Same |
| 3 | GCKR | glucokinase regulatory protein | Same | Downregulated | Downregulated | Same |
| 4 | HK | hexokinase 1 | Upregulated | Same | Same | Same |
| 5 | PFKM | phosphofructokinase, muscle | Same | Downregulated | Same | Same |
| 6 | PFKL | phosphofructokinase, liver | Upregulated | Downregulated | Same | Same |
| 7 | PKM | pyruvate kinase, muscle | Same | Downregulated | Downregulated | Same |
| 8 | PKL | pyruvate kinase, liver |  |  |  | Same |
| 9 | PDH | pyruvate dehydrogenase E1 alpha 1 | Upregulated | Upregulated | Downregulated | Slightly Dn |
| 10 | P-PDH | phospho (Ser 293) pyruvate dehydrogenase E1 alpha 2 | Upregulated | Downregulated | Downregulated | Slightly Dn |
| 11 | P-PDH/PDH ratio | phopshorylation is inhibitory | Same | Reduced greatly | Same | Same |
| 12 | PCX | pyruvate carboxylase | Same | Downregulated | Upregulated | Same |
| 13 | PDK4 | pyruvate dehydrogenase kinase, isoenzyme 4 | Downregulated | Downregulated | Downregulated | Downregulated |
| 14 | PDK1 | pyruvate dehydrogenase kinase, isoenzyme 1 |  | Downregulated |  |  |
| 15 | FOXO3a | forkhead box O3 | Same | Upregulated | Same | Same |
| 16 | P-FOXO3a | phospho (Ser 294) forkhead box O3 | Upregulated | Upregulated | Downregulated | Same |
|  | **Beta oxidation** | |  |  |  |  |
| 17 | HSL | lipase, hormone sensitive | Same | Same |  |  |
| 18 | P-HSL | phospho (Ser 563) lipase, hormone sensitive | Same | Upregulated |  | Downregulated |
| 19 | CPT2 | carnitine palmitoyltransferase 2 | Upregulated | Downregulated | Same | Downregulated |
| 20 | ACADVL | acyl-Coenzyme A dehydrogenase, very long chain | Upregulated | Downregulated | Same | Downregulated |
| 21 | MCAD | acyl-Coenzyme A dehydrogenase, medium chain | Upregulated | Downregulated | Same | Downregulated |
| 22 | ETFA | electron transferring flavoprotein, alpha polypeptide | Upregulated | Upregulated | Downregulated | Downregulated |
| 23 | ACAA1 | acetyl-Coenzyme A acyltransferase 1A, thiolase | Same | Downregulated | Same |  |
| 24 | ACAA2 | acetyl-Coenzyme A acyltransferase 2 (mitochondrial 3-oxoacyl-Coenzyme A thiolase) | Upregulated | Upregulated | Same | Downregulated |
|  | **General energy metabolism** | |  |  |  |  |
| 25 | Shc | src homology 2 domain-containing transforming protein | Downregulated | Downregulated | Downregulated | Downregulated |
| 26 | P(Y239/240)Shc | phospho (Tyr 239/240) src homology 2 domain-containing transforming protein |  | Downregulated |  |  |
| 27 | P(Y317)Shc | phospho (Tyr 317) src homology 2 domain-containing transforming protein |  | Downregulated |  |  |
| 28 | Akt | thymoma viral proto-oncogene, Akt, PKB, PKB/Akt, PKBalpha | Same | Same | Same | Same |
| 29 | P-Akt | phospho (Ser 473) Akt | Same | Upregulated | Same | Same |
| 30 | ERK | p44/42 MAPK (Erk1/2) | Same | Same | Same | Same |
| 31 | P-ERK | phospho (Thr 202/Tyr 204) p44/42 MAPK (Erk1/2) | Downregulated | Downregulated | Same | Downregulated |
| 32 | P(T172)AMPK | phopsho (Thr 172) protein kinase, AMP-activated, alpha 1 catalytic subunit | Upregulated | Downregulated | Downregulated | Same |
| 33 | ME1 | malic enzyme 1, NADP(+)-dependent, cytosolic |  | Downregulated |  |  |
| 34 | MnSOD | manganese SOD, manganese superoxide dismutase | Upregulated | Downregulated | Same | Downregulated |
| 35 | CytC | cytochrome c | Upregulated | Downregulated | Same | Same |
| 36 | GDH | glutamate dehydrogenase |  | Downregulated |  |  |
| 37 | UCP1 | uncoupling protein 1 | Upregulated | Upregulated | Same | Same |
| 38 | UCP2 | uncoupling protein 2 |  |  |  | Same |
| 39 | UCP3 | uncoupling protein 3 | Upregulated |  | Same | Same |
| 40 | VDAC | voltage-dependent anion channel 1 | Same | Downregulated | Same | Same |

Table S1
